# Editorial: Diabetic wound: Multifaceted mechanisms and future of diabetic wound healing

**DOI:** 10.3389/fendo.2022.1068921

**Published:** 2023-01-09

**Authors:** Shiying Shao, Xingwu Ran, Jibiao Li

**Affiliations:** ^1^ Tongji Hospital, Tongji Medical College, Huazhong University of Science and Technology, Wuhan, China; ^2^ West China Hospital, Sichuan University, Chengdu, Sichuan, China; ^3^ School of Biological Sciences, College of Sciences, Georgia Institute of Technology, Atlanta, GA, United States

**Keywords:** diabetic foot ulcer, fibroblast growth factor, adipose-derived stem cell, extracellular vesicle, circular RNA, diabetic foot infection

Diabetic foot ulcer (DFU) is one of the most serious and costly complications of diabetes, a global prevalence of which is 6.3% (5.4-7.3) (1). It is estimated that up to 25% of diabetic patients will develop a foot ulcer in their lifetime (2). DFU could lead to severe morbidity, amputation, and mortality. About 20% of moderate or severe diabetic foot infections results in minor or major amputation (3, 4). In addition, the management of DFU causes a huge burden on socioeconomics and public health.

The standard care of DFU includes blood glucose and infection control, wound dressing changes, surgical debridement, wound off-loading, revascularization, and foot care education (5). However, the therapeutic efficiency of DUF is not satisfying (6, 7) with the median healing time without surgery is 12 weeks (8). In addition, the recurrence rate is as high as 40% within 1 year after ulcer healing (1). Accordingly, researchers have been working to develop efficient and economic adjunctive strategies for DFU treatment.

In recent years, it has been identified that many growth factors play an essential role of wound repair including fibroblast growth factor (FGF), platelet-derived growth factor (PDGF), erythropoietin (EPO), and epidermal growth factor (EGF). Liu et al. give a comprehensive review of the underlying mechanisms of FGF subtypes (e.g., FGF-1, FGF-2, FGF-4, FGF-7, FGF-21 and FGF-23) related to DFU and their potential therapeutic targets. The authors also point out the limitations of FGFs for the treatment of DFU. These growth factors generally have a short half-life, thus requiring repeated administration. In addition, the constant proteolytic environment within chronic wounds could cause the degradation of these growth factors. Improved carriers or delivery methods should be developed for the widely application of FGFs.

Recently, stem cell is widely investigated in the field of wound treatment (9, 10). Stem cell is usually divided into several types including bone marrow-derived stem cell, adipose-derived stem cell (ASC), umbilical cord blood-derived stem cell, and placenta-derived stem cell (11). Liu et al. summarized the mechanisms of ASC about diabetic wound healing, including the promotion of immunomodulation, neovascularization, and fibrogenesis. The administration route of ASC includes direct injection, topical gel treatment, and engineered skin graft sheet. Physicians should make an evaluation to choose appropriate delivery method according to the clinical state of wound. Of note, ASC has the potential of differentiation and a risk of tumorigenesis in ASC-treated patients should be noted. Whether ASC treatment can be considered as a safe method still requires a more longer follow-up period.

Furthermore, adipose tissue is recently identified as an essential endocrine organ which could secrete extracellular vesicles (EVs). EVs contain abundant content such as non-coding RNAs, proteins, and lipids. ASC-EVs play an important role in the process of wound repair and tissue regeneration. Deng et al. point out that ASC-EVs could reduce inflammatory cytokines, prevent cell senescence, increase capillary density, stimulate fibroblasts proliferation and collagen secretion to promote wound repair. Although ASC-EVs have great potential, there are many obstacles. The preparation of ASC-EVs is time-consuming and complicated. Additionally, the extraction quantity of EVs is small and existing extraction schemes fail to meet the clinical standards. Accordingly, a safe and efficient approach of ASC-EVs preparation needs to be developed.

Circular RNAs (circRNAs) are endogenous biomolecules, which exert essential biological functions by acting as microRNA (miRNA) or protein inhibitors (12). Evidences demonstrate that circRNAs could function as competitive endogenous RNA (ceRNAs), which are significantly associated with the onset and development of DFU. A ceRNA network is constructed with 20 differential expression circRNA (DEcircRNAs), 11 differential expression microRNAs (DEmiRNAs), and 9 differential expression mRNAs (DEmRNAs) in the study performed by Zeng et al. They identify that some ceRNAs (JUNB, GATA3, hsa-circ-0049271 and hsa-circ-0074559) might be related to DFU infectious inflammation.

Of note, nearly 50% of patients with DFU are predicted to develop foot infections (13). Du et al. investigate the microbial spectrum isolated from foot ulcers among diabetic patients in China, which may help clinicians choose optimal antibiotics empirically. The authors demonstrate that the microbial infection of foot ulcers among diabetic patients in China is diverse. The microbial spectrum is various in different geographic regions. Amongst, staphylococcus aureus is the predominant bacteria. This study is valuable in guiding the empirical use of antibiotics for diabetic foot infections.

The original and review articles published in this Research Topic provide new insights on DFU treatment. The main challenge of these strategies before widely clinical application is the quality of clinical evidences. Many clinical trials lack double-blind and adequate sample sizes. More well designed RCTs with larger sample size and longer follow up are needed to provide stronger evidences. Here, we sincerely thank all contributors and reviewers for their commitments in this Research Topic and we hope the collection of articles could help readers in their research and clinical work.

## Author contributions

All authors listed have made a substantial, direct, and intellectual contribution to the work and approved it for publication.
